# Related Factors of Insulin Resistance in Korean Children: Adiposity and Maternal Insulin Resistance

**DOI:** 10.3390/ijerph8124596

**Published:** 2011-12-12

**Authors:** Young-Gyu Cho, Jae-Heon Kang, Yang-Im Hur, Jihyun Song, Kang-Sook Lee

**Affiliations:** 1 Department of Family Medicine, Seoul-Paik Hospital, College of Medicine, Inje University, 85 Jeo-dong 2-ga, Jung-gu, Seoul 100032, Korea; Email: jacobel@hanmail.net (Y.-G.C.); fmleader@nuri.net (J.-H.K.); yangimhur@gmail.com (Y.-I.H.); 2 Institute of Clinical Nutrition, Inje University, 85 Jeo-dong 2-ga, Jung-gu, Seoul 100032, Korea; 3 Division of Metabolic Diseases, Center for Biomedical Sciences, National Institute of Health, 187 Osongsaengmyeong 2-ro, Gangoe-myeon, Cheongwon-gun, Chungcheongbuk-do 363951, Korea; Email: jhsong10@korea.kr; 4 Department of Preventive Medicine, College of Medicine, The Catholic University of Korea, Banpo-dong, Seocho-gu, Seoul 137701, Korea

**Keywords:** insulin resistance, child, parent, adiposity, lifestyle habit

## Abstract

Increased adiposity and unhealthy lifestyle augment the risk for type 2 diabetes in children with familial predisposition. Insulin resistance (IR) is an excellent clinical marker for identifying children at high risk for type 2 diabetes. This study was conducted to investigate parental, physiological, behavioral and socio-economic factors related to IR in Korean children. This study is a cross-sectional study using data from 111 children aged 7 years and their parents. Homeostasis model assessment of insulin resistance (HOMA-IR) was calculated using fasting glucose and insulin level as a marker of IR. All children’s adiposity indices (*r* = 0.309–0.318, all *P*-value = 0.001) and maternal levels of fasting insulin (*r* = 0.285, *P*-value = 0.003) and HOMA-IR (*r* = 0.290, *P*-value = 0.002) were positively correlated with children’s HOMA-IR level. There was no statistical difference of children’s HOMA-IR level according to children’s lifestyle habits and socioeconomic status of families. An increase of 1 percentage point in body fat was related to 2.7% increase in children’s HOMA-IR (*P*-value < 0.001) and an increase of 1% of maternal level of HOMA-IR was related to 0.2% increase in children’s HOMA-IR (*P*-value = 0.002). This study shows that children’s adiposity and maternal IR are positively associated with children’s IR.

## 1. Introduction

The prevalence rate of childhood obesity has been increasing throughout the world in developed countries as well as low- and middle-income countries. What is worse is that the relative increasing rate of childhood obesity is faster than that of adult obesity [1,2]. As childhood obesity has increased, many children have been exposed to the risk of metabolic complications of obesity including type 2 diabetes [3]. In addition, type 2 diabetes in Asia has been rapidly increasing in recent decades and is characterized by onset at younger age and lower body mass index (BMI) compared to in the West [4]. Insulin resistance (IR) is an early indicator of impaired glucose metabolism in type 2 diabetes [5] and pre-teen IR can predict future impaired fasting glucose and type 2 diabetes [6]. Therefore, IR is an excellent clinical marker for identifying high risk children for diabetes prevention interventions. Homeostasis model assessment of insulin resistance (HOMA-IR) has been reported to be reliable and valid as a simple and practical surrogate marker to assess IR among children and adolescents [7,8,9]. 

Several factors including family history of type 2 diabetes and IR have been identified as risk factors of IR among children and adolescents [10]. Pankow *et al*. [11] reported that IR was greater in children of parents with type 2 diabetes or insulin resistance syndrome (IRS). Sex [12], race [13], pubertal stage [14] and degree of adiposity [15] have been studied as demographic and physiological factors related to IR in children. It has been also reported that lifestyle factors like physical activity [16] and fruit intake [17] and socio-economic factors like parental education level [18] are associated with IR in children. However, these results have been derived from studies on the Western children. To our knowledge, there have been few studies about related factors of IR in Asian children.

This study was conducted to investigate parental, physiological, behavioral and socio-economic factors related to IR in Korean children.

## 2. Methods

### 2.1. Subjects

Between October and November 2005, 124 children (1st grade students) and their parents were recruited from 7 elementary schools in Gwacheon and Seoul. They were invited to join this family study through phone calls and letters. Gwacheon lies adjacent to the southern part of Seoul and inhabitants of the city have similar socio-economic status with those of Seoul [19]. Two children, whose fathers were taking oral medication for previously diagnosed diabetics, six children, who did not appropriately answer to questionnaires about lifestyle habits and five children, whose levels of fasting insulin was missing were excluded. Finally, the data of 111 children (67 boys and 44 girls) and their parents were available for the final analyses. This study protocol was approved by the Institutional Review Board of Seoul-Paik Hospital, Inje University. Informed consent was obtained from the children and their parents.

### 2.2. Measurements

Anthropometric measurements were performed in the morning after fasting above 12 hours with light clothing without shoes by trained nurses under surveillance of investigators of this study. Each anthropometric measurement was done by the same nurse, the same instrument and the same technique in order to reduce variance of each measurement. Height was measured through an automatic height scale (DS-102, Jenix, Seoul, Korea). A bioelectrical body composition analyzer (BC-418, TANITA, Tokyo, Japan) was used for measurement of body weight and body fat percentage (BF%). Waist circumference was measured at the end of normal expiration, at the level of the midpoint between lower border of 12th rib and upper border of iliac crest using a non-elastic tape. BMI was calculated by dividing body weight (kg) by height^2^ (m). 

Fasting blood samples for measurements of plasma glucose and insulin were obtained from antecubital vein. The glucose concentration was determined with hexokinase method by an auto-analyzer (Hitachi 736-40, Hitachi Co., Tokyo, Japan). The insulin concentration was measured by radioimmunoassay kit (Diagnostic Products Corporation, Los Angeles, CA, USA). HOMA-IR was calculated using fasting glucose level and fasting insulin level according to the following formula: HOMA-IR = fasting insulin (μU/mL) × fasting glucose (mmol/L)/22.5 [20]. 

Information about socioeconomic status (SES) of families and birth weight and lifestyle habits of children was obtained by self-administered questionnaires. Questionnaires were separately made for children and for parents. Parents were asked to help their children to fill out questionnaires for children. Questionnaires for children’s dietary habits were composed of intake frequencies of fruits, vegetables and fast-foods. For children’s physical activity habits, frequencies of moderate physical activity and vigorous physical activity and daily screen time were inquired. Moderate physical activity and vigorous physical activity were defined as physical activity that was of slightly faster breathing rate than usual life and last at least 30 minutes and physical activity that was quite breathless and sweating, and last at least 20 minutes, respectively. Screen time was defined as time watching television or using computer for game or internet. Parental education level, total household income and maternal working status were investigated as markers of SES of families.

### 2.3. Statistical Analyses

Age, anthropometric variables, fasting glucose, fasting insulin and HOMA-IR of children and their parents were managed as continuous variables and sex, birth weight and lifestyle habits of children and markers of SES of families were managed as categorical variables. Fasting insulin and HOMA-IR were analyzed after log transformation because they did not have a normal distribution. The relationships between children’s levels of fasting glucose, fasting insulin and HOMA-IR and other continuous variables were assessed by sex-adjusted partial correlation analyses. Two-way ANOVA was used to compare sex-adjusted mean levels of these indices according to categorical variables. Multiple regression analyses were conducted to identify factors that were independently related to HOMA-IR. Variables that were significantly related to HOMA-IR in partial correlation analysis or two-way ANOVA were included as independent variables in regression models. Because children’s adiposity indices (waist circumference, BMI and BF%) were highly correlated with one another (*r*: 0.889–0.911), regression models with two and more children’s adiposity indices together can lead to multicollinearity. We included only waist circumference among children’s adiposity indices in the main regression model because waist circumference had the highest correlation with HOMA-IR among children’s adiposity indices. For the same reason, neither fasting glucose nor insulin of parents were together included with their HOMA-IRs in the same regression model. For considering differences of adiposity depending on sex, sex was adjusted in these analyses. Finally, Sex, waist circumference and maternal level of HOMA-IR were included as independent variables in the main regression model. We think that because all values of the variance inflation factor (VIF) for independent variables in the main regression models were less than 2, there was no problem from multicollinearity. All statistical analyses were performed with PASW Statistics 18 (SPSS Inc., Chicago, IL, USA) and *P*-values less than 0.05 was considered statistically significant. 

## 3. Results

Children’s mean age was 7.3 ± 0.3 years. Some anthropometric variables such as BMI and waist circumference were higher in boys than girls. However, there were no statistical differences of glucose metabolism indices such as fasting glucose, fasting insulin and HOMA-IR between boys and girls. Children’s mean levels of fasting glucose, fasting insulin and HOMA-IR were 89.1 ± 7.1 mg/dL, 5.04 ± 1.83 μU/mL and 1.11 ± 1.86, respectively (Table 1). Boys reported more frequent moderate and vigorous physical activities compared to girls. The other lifestyle habits were not different between boys and girls (data not shown).

**Table 1:** General characteristics of children. ^a^

	Boys (n = 67)	Girls (n = 44)	Total (n = 111)	*P*-value ^b^
**Age (years)**	7.3 (0.3)	7.2 (0.3)	7.3 (0.3)	0.265
**Body mass index (kg/m^2^)**	18.0 (3.0)	16.5 (2.1)	17.4 (2.8)	0.002
**Body fat percentage (%)**	21.9 (8.4)	20.9 (5.9)	21.5 (7.5)	0.462
**Waist circumference (cm)**	59.6 (7.5)	55.5 (6.4)	58.0 (7.4)	0.004
**Fasting glucose (mg/dL)**	89.8 (7.7)	88.1 (6.0)	89.1 (7.1)	0.209
**Fasting insulin (μU/mL) ^c^**	5.06 (1.68)	5.02 (2.05)	5.04 (1.83)	0.948
**HOMA-IR ^c^**	1.12 (1.71)	1.09 (2.08)	1.11 (1.86)	0.829
**Birth weight**				
** <3.5 kg**	37 (55.2)	28 (65.1)	70 (59.1)	0.303
** ≥3.5 kg**	30 (44.8)	15 (34.9)	46 (40.9)	

^a^ Values are expressed as mean (SD) or n (%). ^b^ *P*-value by t-test or Chi-square test (Boys *vs*. Girls). ^c^ Geometric mean (GSD) computed from log-transformed data.

The mean ages of mothers and fathers were 37.7 ± 3.9 years and 40.5 ± 4.2 years, respectively. Mother’s mean levels of fasting glucose, fasting insulin and HOMA-IR were 87.6 ± 7.3 mg/dL, 3.40 ± 2.22 μU/mL and 0.73 ± 2.27, respectively. Father’s mean levels of fasting glucose, fasting insulin and HOMA-IR were 94.1 ± 15.7 mg/dL, 10.17 ± 2.19 μU/mL and 2.34 ± 2.32, respectively (Table 2). It was investigated that more than 70.0% of parents graduated from college and 34.9% of mothers were employed. In addition, 36.9% of families had a monthly household income over 4 million won (exchange rate in 2005: 1,013 Korean Won for 1 US dollar; data not shown). 

**Table 2:** General characteristics of parents. ^a^

	Mothers	Fathers
**Age (years)**	37.7 (3.9)	40.5 (4.2)
**Body mass index (kg/m^2^)**	22.9 (3.3)	24.7 (3.0)
**Body fat percentage (%)**	33.5 (5.6)	25.3 (5.5)
**Waist circumference (cm)**	77.1 (8.4)	86.5 (7.7)
**Fasting glucose (mg/dL)**	87.6 (7.3)	94.1 (15.7)
**Fasting insulin (μU/mL) ^b^**	3.40 (2.22)	10.17 (2.19)
**HOMA-IR ^b^**	0.73 (2.27)	2.34 (2.32)

^a^ Values are expressed as mean (SD). ^b^ Geometric mean (GSD) computed from log-transformed data.

**Table 3:** Sex-adjusted partial correlation coefficients between indices of glucose metabolism, and adiposity indices of children and glucose metabolism of their parents.

	Fasting glucose		Log fasting insulin		Log HOMA-IR	
	*r*	*P*-value ^a^	*r*	*P*-value ^a^	*r*	*P*-value ^a^
***Children***						
**Waist circumference**	-0.105	0.276	0.338	<0.001	0.318	0.001
**Body mass index**	-0.082	0.394	0.332	<0.001	0.315	0.001
**Body fat percentage**	-0.074	0.440	0.325	0.001	0.309	0.001
***Mothers***						
**Fasting glucose**	0.270	0.004	0.085	0.377	0.120	0.216
**Log fasting insulin**	0.000	1.000	0.291	0.002	0.285	0.003
**Log HOMA-IR**	0.029	0.767	0.292	0.002	0.290	0.002
***Fathers***						
**Fasting glucose**	0.264	0.006	0.071	0.466	0.103	0.290
**Log fasting insulin**	0.077	0.428	0.084	0.388	0.093	0.338
**Log HOMA-IR**	0.124	0.200	0.089	0.357	0.104	0.282

^a^ *P*-value by partial correlation analysis.

Sex-adjusted partial correlation coefficients between children’s glucose metabolism indices, and children’s adiposity indices and glucose metabolism indices of their parents were presented in Table 3. All children’s adiposity indices such as waist circumference, BMI and BF% were positively correlated with children’s levels of fasting insulin (*r* = 0.325–0.338, all *P*-value < 0.01) and HOMA-IR (*r* = 0.309–0.318, all *P*-value < 0.01). Whereas maternal levels of fasting insulin and HOMA-IR have significant positive correlation with children’s level, no significant correlation of levels of fasting insulin and HOMA-IR between fathers and children was not observed. Children’s fasting glucose level had no statistical significant correlations with any of children’s adiposity indices. However, children’s fasting glucose level were positively correlated with parental fasting glucose levels (mothers: *r* = 0.270, fathers: *r* = 0.264; both *P*-value < 0.01). Significant correlations between parental levels of adiposity indices and children’s levels of fasting insulin and HOMA-IR were not observed (data not shown).

**Table 4:** Sex-adjusted mean levels of fasting glucose, fasting insulin, and HOMA-IR according to lifestyle habits. ^a^

	Fasting glucose (mg/dL)	Fasting insulin (μU/mL) ^b^	HOMA-IR ^b^
**Intake of fruits**			
**≥7 times/week (n = 49)**	89.1 (1.0)	5.01 (1.09)	1.10 (1.09)
**4–6 times/week (n = 34)**	88.4 (1.2)	5.09 (1.11)	1.11 (1.11)
**≤3 times/week (n = 28)**	89.4 (1.4)	5.03 (1.13)	1.11 (1.13)
***P*-value ^c^**	0.832	0.994	0.998
**Intake of fresh vegetables**			
**≥7 times/week (n = 55)**	88.4 (1.0)	5.20 (1.09)	1.13 (1.09)
**4–6 times/week (n = 21)**	89.1 (1.6)	4.72 (1.15)	1.04 (1.15)
**≤3 times/week (n = 35)**	89.8 (1.2)	4.97 (1.11)	1.10 (1.11)
***P*-value ^c^**	0.660	0.883	0.868
**Intake of fast-foods**			
**<1 times/week (n = 61)**	89.2 (0.9)	5.33 (1.08)	1.17 (1.08)
**≥1 times/week (n = 50)**	88.6 (1.0)	4.70 (1.09)	1.02 (1.09)
***P*-value ^c^**	0.663	0.284	0.277
**Moderate physical activity**			
**≥5 days/week (n = 31)**	88.4 (1.3)	5.28 (1.12)	1.15 (1.12)
**2–4 days/week (n = 52)**	88.2 (1.0)	4.66 (1.09)	1.01 (1.09)
**≤1 days/week (n = 28)**	90.7 (1.3)	5.49 (1.12)	1.23 (1.13)
***P*-value ^c^**	0.327	0.462	0.395
**Vigorous physical activity**			
**≥5 days/week (n = 32)**	87.6 (1.3)	4.48 (1.12)	0.96 (1.12)
**2–4 days/week (n = 44)**	88.8 (1.1)	5.14 (1.10)	1.12 (1.10)
**≤1 days/week (n = 35)**	90.1 (1.2)	5.40 (1.11)	1.20 (1.11)
***P*-value ^c^**	0.386	0.457	0.376
**Screen time**			
**<60 minutes/day (n = 40)**	87.6 (1.1)	5.08 (1.10)	1.10 (1.11)
**60–119 minutes/day (n = 38)**	91.0 (1.2)	4.60 (1.11)	1.03 (1.11)
**≥120 minutes/day (n = 29)**	88.0 (1.3)	5.61 (1.12)	1.22 (1.13)
***P*-value^c^**	0.082	0.430	0.576

^a^ Values are expressed as sex-adjusted mean (SE). ^b^ Geometric mean (GSD) computed from log-transformed data. ^c^ *P*-value by two-way ANOVA.

Sex-adjusted mean levels of fasting glucose, fasting insulin and HOMA-IR according to children’s lifestyle habits were shown in Table 4. Children’s lifestyle habits related with fasting insulin or HOMA-IR was not identified. In addition, there were no statistical differences of mean levels of fasting glucose, fasting insulin and HOMA-IR according to SES of families and children’s birth weight (data not shown). 

Multiple regression analyses were conducted to identify factors that were independently related to children’s HOMA-IR. Sex, waist circumference and maternal level of HOMA-IR were included as independent variables in the main regression model. Children’s waist circumference and maternal level of HOMA-IR had significant positive relationships with children’s HOMA-IR level. An increase of 1 percentage point in body fat was related to 2.7% increase in children’s HOMA-IR (*P*-value < 0.001) and an increase of 1% of maternal HOMA-IR level was related to 0.2% increase in children’s HOMA-IR (*P*-value = 0.002) (Table 5). In case waist circumference or BMI was in place of BF% in a regression model, there was no change of the results (data not shown). These results were also consistent in analysis where fasting insulin level was substituted as dependent variable for level of HOMA-IR (data not shown). 

**Table 5:** Results of multiple regression analysis on log HOMA-IR.

	*β*	SE	*P*-value
**Constant**	-1.345	0.414	0.002
**Sex**			
** Girls**			
** Boys**	-0.043	0.110	0.693
**Waist circumference**	0.027	0.007	<0.001
**Mother log HOMA-IR**	0.202	0.063	0.002

## 4. Discussion

This study aimed to investigate which factors were associated with IR in Korean children using cross-sectional data from 111 children aged 7 years and their parents. We thought that parental, physiological, behavioral and socio-economic factors have their own role for the development of IR in children. This study shows that maternal IR and amount of adiposity have significant independent relationships with children’s IR.

There are a lot of evidences that impaired glucose metabolism begins early in life [21,22]. However, fasting glucose in children may not be a proper clinical marker because impaired fasting glucose is a late indicator of impaired glucose metabolism [23] and fasting glucose in children has remarkably narrow distribution [24]. Significant difference of fasting glucose level according to amount of adiposity or lifestyle habits is not observed in this study, as well. On the contrary, IR in children is an early indicator of impaired glucose metabolism [5] and a predictor of future impaired fasting glucose and type 2 diabetes [6]. Various methods were developed for assessing IR including the hyper-insulinemic-euglycemic clamp [25] and the frequently sampled intravenous glucose tolerance test [26]. However, these methods are too invasive, complicated and expensive for epidemiologic study and routine clinical assessment of children. HOMA-IR was developed as a simple and practical surrogate marker to assess IR [20] and has been proved to be reliable and valid among children and adolescents in several studies [7,8,9]. 

This study shows that HOMA-IR level is higher in children whose mothers have higher HOMA-IR level. Children’s IR is known to have familial background [27]. Children of parents with type 2 diabetes or IRS have higher IR [11]. There are a lot of study results on heritability of HOMA-IR or fasting insulin. Although there is a little difference of study results according to study population and methods, it has been reported that heritability of HOMA-IR or fasting insulin is from 0.23 to 0.54 [28,29,30,31,32,33]. In this study, only IR of mothers among two parents had the significant relationship with children’s IR and IR of fathers was not related to children’ IR. This difference in the relationship with children’s IR between IRs of both parents could not be explained by genetics. Freeman *et al*. [28] reported that childhood household environment significantly contribute to variance of HOMA-IR level in adulthood. Parents share not only genes but also environments with their children. As mothers primarily bring up their children in the Korean society, they might share more household environment and health behaviors with their children. The stronger linkage between mothers and children could be an important cause that maternal IR was different from paternal IR in the relationship with children’s IR. 

All children’s adiposity indices such as waist circumference, BMI and BF% are positively correlated with children’s HOMA-IR level in this study. This result is consistent with previous studies. Previous studies have reported that obesity is one of the most important determinants of IR in children. Lee *et al*. [12] investigated the distribution and determinants of HOMA-IR among U.S. adolescents who participated in the National Health and Nutrition Examination Survey 1999–2002. They reported that obese children have significantly higher HOMA-IR level compared to normal weight children (mean HOMA-IR level: 4.93 *vs*. 2.30). Young-Hyman *et al*. [34] demonstrated that HOMA-IR is positively correlated with amount of adiposity in 5–10 years old overweight children. Additionally, Gardner *et al*. [35] showed that adiposity at 5 years can predict HOMA-IR at 8 years, especially in girls.

We could not find children’s lifestyle habits related to children’s HOMA-IR in this study. It is well known that regular physical activity can improve insulin sensitivity [36]. It is recommended that at least 60–90 minutes of daily moderate-vigorous physical activity is required for prevention and management of type 2 diabetes in youth [37]. Even though children’s levels of fasting insulin and HOMA-IR decreased with increase of vigorous physical activity frequency in this study, this was not statistically significant. This insignificant result could be because of inaccuracy of physical activity measurement. We measured physical activity not by objective tools like accelerometers but by self-administered questionnaires. Although it was well studied that unhealthy eating habits are associated with adiposity, an important determinants of IR in children [38], there are few studies about their direct relationship with children’s IR. Lindquist *et al.* [17] reported that fruit and carbohydrate intakes are positively associated with insulin sensitivity and vegetable intake is negatively associated with acute insulin response in African American and white children. However, we could not identify any relationship between children’ eating habits and HOMA-IR. We think that further research is needed to examine dietary factors associated with IR in children.

We reported a result on the relationship between SES of families and children’s overweight in the Korean society in a previous study [39]. The study showed that low maternal education level was related to overweight of children aged 8 years in Korean society. Because overweight is one of the most important determinants of IR in children, we expected to identify the relationships between children’s IR and SES of families including maternal education level in this study. However, we could not find any relationship between SES of families and children’s HOMA-IR in this study. There were few previous studies on the relationship between SES of families and children’s IR. Contrary to our results, Goodman *et al*. [18] reported that lower parent education was associated with higher IR in adolescents aged 12–19 years. It is difficult to compare the results of both studies because participants of both studies are different in ages. The relationships between parental education level and children’s IR could be changing depending on children’s ages and the influence of parental education level on children’s IR could appear later than that on children’s adiposity.

This study has several limitations to be considered. Cross-sectional design of this study prevented causal inferences. We measured IR not by more precise clamp studies, but by HOMA-IR. Clamp studies cannot be used because of invasiveness, cost and feasibility. HOMA-IR was proved to correlate with more precise measures of IR [7,8,9]. This study is also limited because children’s lifestyle habits were assessed only by self-administered questionnaires. Even though someone can be interested in which factors had the largest influence on children’s IR, this study could not reveal exactly the extent that each factor affected children’s IR. In spite of these several limitations, this study has advantages in directly measuring adiposity and HOMA-IR in both children and their parents. To our knowledge, this study is the first study that investigates parental, physiological, behavioral and socio-economic factors associated with IR in Asian children.

## 5. Conclusions

Increased adiposity makes type 2 diabetes develop in children with familial predisposition in younger age. We demonstrate that adiposity and maternal IR are independently associated with children’s IR. Prevention and management of obesity could be practical targets for prevention of type 2 diabetes in Korean children.
